# Impact of structural aberrancy of polysialic acid and its synthetic enzyme ST8SIA2 in schizophrenia

**DOI:** 10.3389/fncel.2013.00061

**Published:** 2013-05-07

**Authors:** Chihiro Sato, Ken Kitajima

**Affiliations:** Laboratory of Animal Cell Function, Bioscience and Biotechnology Center, Nagoya UniversityNagoya, Japan

**Keywords:** schizophrenia, psychiatric disorder, polysialic acid, polysialyltransferase, ST8SIA2, NCAM, BDNF, dopamine

## Abstract

Psychiatric disorders are a group of human diseases that impair higher cognitive functions. Whole-genomic analyses have recently identified susceptibility genes for several psychiatric disorders, including schizophrenia. Among the genes reported to be involved in psychiatric disorders, a gene encoding a polysialyltransferase involved in the biosynthesis of polysialic acid (polySia or PSA) on cell surfaces has attracted attention for its potential role in emotion, learning, memory, circadian rhythm, and behaviors. PolySia is a unique polymer that spatio-temporally modifies neural cell adhesion molecule (NCAM) and is predominantly found in embryonic brains, although it persists in areas of the adult brain where neural plasticity, remodeling of neural connections, or neural generation is ongoing, such as the hippocampus, subventricular zone (SVZ), thalamus, prefrontal cortex, and amygdala. PolySia is thought to be involved in the regulation of cell-cell interactions; however, recent evidence suggests that it is also involved in the functional regulation of ion channels and neurologically active molecules, such as Brain-derived neurotrophic factor (BDNF), FGF2, and dopamine (DA) that are deeply involved in psychiatric disorders. In this review, the possible involvement of polysialyltransferase (ST8SIA2/ST8SiaII/STX/Siat8B) and its enzymatic product, polySia, in schizophrenia is discussed.

## Introduction

Schizophrenia is a severe and persistent psychiatric disorder that affects approximately one percent of the population worldwide. Although several factors are associated with an increased risk of developing schizophrenia, the underlying mechanism for this disorder remains unclear. The overall risk profile is mainly determined by the presence of causative genes, such as those encoding disrupted-in-schizophrenia 1 (DISC1) (Millar et al., 2001), Neuregulin 1 (Stefansson et al., 2002), COMP (catechol-*o*-methyltransferase) (Strous et al., 1997), and dopamine (DA)-receptors (Allen et al., 2008). Schizophrenia is also deeply related to neurodevelopmental and neurodegenerative diseases involving disconnectivity and disorder of synapses (Woods, 1998; Ashe et al., 2001), which may occur at a restricted stage during early brain development. Despite the identification of these schizophrenia risk factors, studies investigating the specific relationships between genes and phenotypes are needed. In this regard, the contribution of the posttranslational modification of proteins and the presence of membrane glycans, such as glycoproteins, gangliosides, and proteoglycans, are important to consider. However, these factors are often overlooked, even though nearly all cell-surface and extracellular proteins are glycosylated.

Animal cells are covered by the glycocalyx, a dense sugar coat composed of glycoproteins, glycolipids, and proteoglycans that provide hot spots for interaction with other cells and extracellular space components. The glycocalyx has a unique and characteristic structure, particularly in the peripheral region, which is the dominant region for communication with other cells and extracellular factors. Among glycans in the glycocalyx, polysialic acid (polySia, PSA), HNK-1, and sulfated glycans share spatio-temporally regulated expression. In particular, polySia is of considerable recent interest in the field of schizophrenia research, and has been studied using histochemical, genome-wide, and biochemical approaches.

In this review, we describe: (1) the unique features of polySia and its biosynthesis enzyme, ST8SIA2; (2) the newly discovered functions of polySia; and (3) the relationship between polySia and psychiatric disorders, particularly schizophrenia, which have been reported to date.

## Structure and distribution of polySia and ST8SIA2

**Figure 1:**
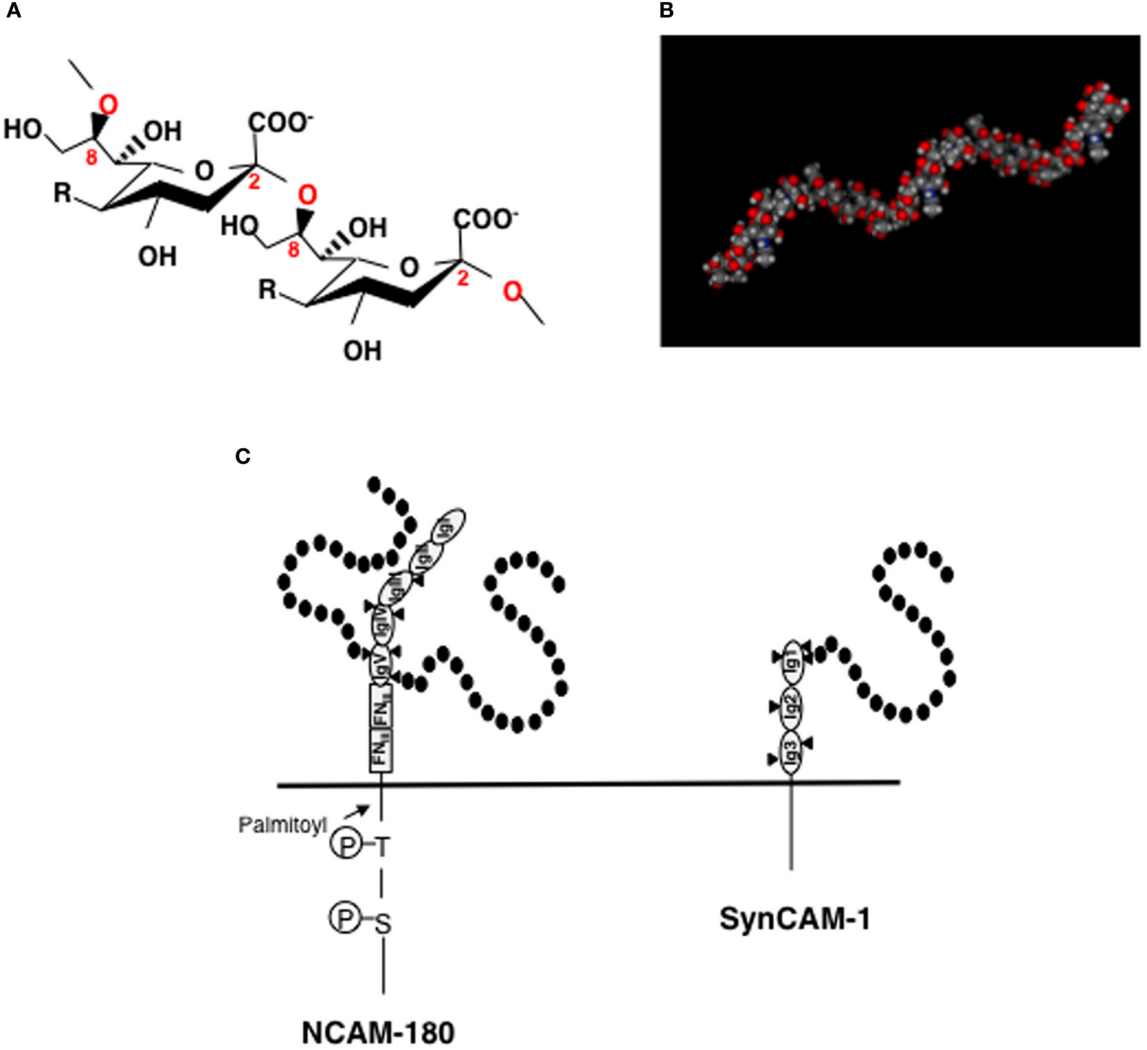
**Structures of polySia, polySia-NCAM, and polySia-synCAM. (A)** α2-8linked polySia structure. R = -NHCOCH_3_, Neu5Ac; -NHCOCH_2_OH, Neu5Gc, -OH, KDN. In the brain, polySia is polyNeu5Ac. **(B)** Molecular modeling of polySia (DP = 12). The polySia structure is linked to Gal at the C3-position. Calculated α2,8-linked dodecaNeu5Ac-Gal structure is shown as space-filling models. The MOE program was used for the construction and calculation of the energies of α2,8-linked polySia. **(C)** polySia-NCAM-180 and polySia-synCAM-1. **Left panel**, NCAM-180. NCAM has five immunoglobulin domains (IgI~IgV) and two fibronectin type-III (FN_III_) domains in its extracellular domain. In the IgV domain, two of the three *N*-glycosylation sites (triangle) are polysialylated and are indicated by black circles (sialic acid). **Right panel**, SynCAM-1. SynCAM-1 has three Ig domains. In the IgI domain, one of the three *N*-glycosylation sites (triangle) is occupied by polySia which is indicated by black circles (sialic acid).

PolySia is a linear homopolymer of sialic acid with a degree of polymerization (DP) ranging from 8 to 400 (Figure 1A) (Troy, 1996) and was first found as part of polysaccharide chains on the cell surface of neuroinvasive bacteria. PolySia is thought to adopt a helical structure (Evans et al., 1995) (Figure 1B) and can be identified with specific probes, such as antibodies (mAb. 735 and mAb. 12E3) and endo-*N*-acylneuraminidase (Endo-N) (Troy, 1996; Rutishauser, 2008; Sato, 2013). The expression of polySia in vertebrates is spacio-temporally regulated (Rutishauser, 2008) and is highly restricted to the brain during embryonic and post-neonatal development. In the adult brain, polySia is typically found at very low levels; however, it persists in distinct regions where neural plasticity, remodeling of neural connections, or neural generation is ongoing, such as the hippocampus, subventricular zone (SVZ), thalamus, prefrontal cortex, and amygdala. It is also interesting that polySia expression is restricted to interneurons in different cortical regions, such as the prefrontal cortex and amygdala (Gómez-Climent et al., 2011; Nacher et al., 2013). There are several precise reviews on polySia and polysialylated NCAM (polySia-NCAM) expression in brains (Bonfanti, 2006; Nacher et al., 2013; Sato, 2013). The major carrier protein of polySia in vertebrate brains is neural cell adhesion molecule (NCAM) (Finne et al., 1983). As NCAM expression levels remain relatively unchanged throughout normal development, it is speculated that polySia expression is tightly correlated with that of the polySia biosynthetic enzymes, particularly the polysialyltransferases ST8SIA2 and/or ST8SIA4 (Mühlenhoff et al., 2009). Recently, ST8SIA2 was also demonstrated to modify synaptic cell adhesion molecule 1 (synCAM-1) (also known as Cadm1 or TSLC1) (Rollenhagen et al., 2012). The polysialylation sites on NCAM and synCAM have been well studied. NCAM consists of five immunoglobulin (Ig)-like domains with six *N*-glycosylation sites and two fibronectin type-III (FN_III_)-like -domains in the extracellular region. PolySia chains are linked to the tri- or tetra-antennary *N*-linked glycan chains on Ig domain V of NCAM (Figure 1C, left panel). First FN_III_ domain is important for the polysialylation of NCAM (Close et al., 2003). In SynCAM, which consists of three extracellular Ig domains, polySia is linked to a *N*-linked glycan chain on Ig domain I (Figure 1C, right panel).

**Figure 2:**
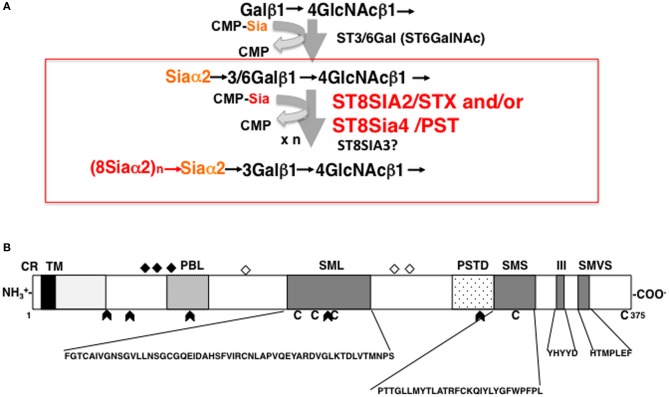
**Biosynthesis of polySia and schematic structure of ST8SIA2. (A)** Mono and di/oligo/polysialylation pathway on the terminal Gal residue of N-glycan chains. Asialo-glycoprotein is mono-sialylated by the action of ST3/6GAL. ST8SIA2, ST8SIA4, and/or ST8SIA2/4 can synthesize polySia chains on the monosialyl residue, particularly on NCAM. **(B)** Schematic representation of the protein structure of ST8SIA2. ST8SIA2 consists of a short cytoplasmic region (CR) connected to a transmembrane (TM) region, which is followed by an intraluminal region consisting of a stem region and catalytic domain. In the catalytic domain, SML (Sialyl Motif Large), SMS (Small), motif III, and SMVS (Very Short) are present. Two additional regions have been newly characterized: a polybasic polysialyltransferase domain (PSTD) and a polybasic motif named the polybasic region (PBR). C, cysteine residues; diamonds, *N*-glycosylation site; open diamonds, autopolysialylation sites (Asn 89, 219, and 234) of six *N*-linked glycans; arrowheads, exon junction.

Both ST8SIA2 and ST8SIA4 catalyze the transfer of sialic acid through α2,8-linkages onto sialic acid residues and cooperatively elongate the polySia chain using CMP-Sia as a donor substrate (Figure 2A). ST8SIA2 and ST8SIA4, which belong to a family of sialyltransferases that are part of the glycosyltransferase family, are type II membrane proteins that localize in the Golgi apparatus. The protein structure of ST8SIA2 and ST8SIA4 consists of a short cytoplasmic region connected to a transmembrane (TM) region that is joined to an intra-luminal region (Figure 2B). The intra-luminal region consists of a stem region and a catalytic domain, and sialyl motifs L (Large), S (Small), III, and VS (Very Short), which are a common feature of α2,3-, α2,6-, and α2,8-sialyltransferases (Angata and Fukuda, 2003; Takashima, 2008). Sialyl motif L is positioned in the center of the enzyme and is characterized by a 55-amino-acid region that serves as a donor substrate (CMP-Sia) binding site. Sialyl motif S is located at the C-terminal region of the enzyme and consists of 28 amino acid residues that are involved in the binding of both donor and acceptor substrates. Sialyl motif VS (HXXXXEX) is also located in the C-terminal region and is reported to be involved in catalytic activity. Histidine (H) and glutamic acid (E) residues in this motif are highly conserved between sialyltransferases. Motif III (YHYYD) is located between sialyl motif S and VS and is also involved in the catalytic activity of ST8SIA2 and ST8SIA4. In addition, a novel polybasic polysialyltransferase domain (PSTD; 32 amino acids) identified next to the motif S in both sialyltransferases was demonstrated to be involved in the polysialylation activity of ST8SIA4 (Nakata et al., 2006). More recently, a second conserved polybasic motif, named polybasic region (PBR), was identified close to sialyl motif L in ST8SIA2 and ST8SIA4 (Foley et al., 2009). The PBR consists of 35 amino acids, of which seven are the basic amino acids arginine (R) and lysine (K), and is involved in NCAM-specific polysialylation. These basic amino acids are considered to be important for IgV specific polysialylation of NCAM through binding via acidic patch of first FNIII domain.

The human ST8SIA2 gene is located on chromosome 15 (Angata et al., 1997) and the corresponding enzyme consists of six exons (Takashima, 2008). Although the promoter region of the ST8SIA2 gene has not been well examined *in vitro*, the schizophrenia-associated haplotype block of ST8SIA2 appears to localize in the putative promoter region based on database searches (TFSEARCH, http://mbs.cbrc.jp/research/db/TFSEARCHJ.html), which identified several putative consensus motifs and transcriptional factor binding sites, including those for CCAAT, MZF1, CREB, GATA, TATA, and SP1 (Arai et al., 2006). In the mouse genome, ST8SIA2 expression is driven by SP1-binding motifs present in a TATA-less GC-rich domain (Yoshida et al., 1996) and by the cAMP-CREB cascade (Nakagawa et al., 2002). It is also reported that ST8SIA2 is under the control of Pax3 (Mayanil et al., 2000, 2001), a member of a paired homeobox family of evolutionary conserved transcription factors that are important for brain development.

## Phenotypes of polySia-impaired mice

To understand the function of polySia, a NCAM-deficient mouse line was established (Cremer et al., 1994), because NCAM is the major carrier of polySia in the brain. Based on the observed phenotype of NCAM knockout (KO) mice, polySia-NCAM was shown to be required for cell migration, neuronal path finding, and synaptic plasticity necessary for memory formation. As polySia-NCAM is highly expressed in suprachiasmatic nuclei (SCN), the effect of polySia on SCN-mediated circadian clock function was analyzed in adult mice. The removal of polySia from SCN by the microinjection of endoneuraminidase (endo-N; polySia specific endo-sialidase) shortened the free-running period to a similar extent as in the NCAM KO mutant, demonstrating that NCAM and polySia are involved in the development and physiology of the mammalian SCN circadian clock (Shen et al., 1997). NCAM-KO mice also exhibit increased anxiety, which is thought to be due to altered serotoninergic transmission (Stork et al., 1999).

To investigate the function of polySia in more detail, ST8SIA2 single KO (SKO) (Angata et al., 2004) and ST8SIA4 SKO mice (Eckhardt et al., 2000) were established. ST8SIA2 SKO mice exhibit misguided infrapyramidal mossy fibers and form ectopic synapses in the hippocampus. Quantification of the myelinated axons in ST8SIA2 SKO mice revealed the number and size of regenerated fibers are significantly decreased, although remyelination is not impaired. In addition, ST8SIA2 SKO mice also exhibit higher exploratory drive and reduced behavioral responses to Pavlovian fear conditioning. The phenotype of ST8SIA4 SKO mice was characterized by a marked decrease of polySia in the CA1 region of Ammon's horn, indicating that ST8SIA4 is involved in hippocampal synaptic plasticity, particularly in long-term potentiation (LTP) and long-term depression (LTD) in the hippocampal CA1 region. Interestingly, both ST8SIA2 and ST8SIA4 SKO mice show a profound impairment in social behavior (Calandreau et al., 2010), including decreased motivation to interact socially. ST8SIA2 SKO mice exhibit a behavioral profile that combines increased aggressive behavior and hyperactivity with reduced anxiety-like behavior, which is similar to certain attention-deficit hyperactivity disorder-related pathologies. In contrast, ST8SIA4 SKO mice are predominantly characterized by decreased motivation in social interaction. This behavior is the result of olfactory deficits and is associated with a clear decrease in polySia-NCAM expression in all brain regions. Recently, precise observations of the polySia-NCAM expression with adult SKO mice especially in cerebral cortex were reported. The facts that ST8SIA4 is a responsible for polySia expression in mature interneurons and in most regions of cortical neuropili and that ST8SIA2 is the main polysialyltransferase in immature neurons of the paleocortex layer II and the hippocampal subgranular zone (Nacher et al., 2010) are important information to understand biological meaning of polysialyltransferases.

As polySia can be biosynthesized by either one of the two polysialyltransferases, SKO mice still contain a large amount of residual polySia in the brain (Oltmann-Norden et al., 2008). Thus, to completely remove the background levels polySia, ST8SIA2, and ST8SIA4 double KO (DKO) mice were established (Weinhold et al., 2005). DKO mice show a severe phenotype, characterized by postnatal growth retardation, precocious death, high incidence of hydrocephalus and agenesis, and hypoplasia of major brain fiber tracts. Because almost all DKO mice die soon after birth (80% die before the age of 4 weeks), it appears that the presence of polySia on not only NCAM, but also other polySia-containing glycoproteins, plays a direct and important role in both brain and other unknown tissue functions. Interestingly, in NCAM, ST8SIA2, and ST8SIA4 triple-KO (TKO) mice, the severe phenotype of the DKO mice is rescued, suggesting that an uncontrolled type of NCAM-mediated cell adhesion is followed by increased signal transduction events. In TKO mice, improved signaling via increased cell-cell interactions in the polySia-deficient brain is likely to result from the reduced levels of cell adhesion molecules resulting from the NCAM deficiency. Thus, the reduction of NCAM leads to the recovery of normal physiological interactions and to the rescue of the severe phenotype of polySia-DKO mice (Hildebrandt et al., 2007).

## Known functions of polySia

It is well known that polySia is involved in numerous important neurological functions, including neural outgrowth, cell migration, axonal guidance, and branching, neuronal pathfinding, lamination of mossy fibers, LTP, and LTD in the hippocampus, synapse formation, and plasticity. The expression of polySia is spatio-temporally regulated and intimately influences neurogenesis and neural circuit development. Thus, the anomalous expression of polySia impairs learning, memory, behavior (fear and social behavior), and circadian clock rhythm. There are several excellent reviews on the role of polySia in different aspects of neural development, plasticity, and repair (Bonfanti, 2006; Gascon et al., 2007; Hildebrandt et al., 2007; Rutishauser, 2008). Although the underlying mechanisms for how aberrant expression of polySia leads to these phenotypic abnormalities remain to be elucidated, several properties, and functions of polySia may shed light on this issue.

### Regulator of cell adhesion

**Figure 3:**
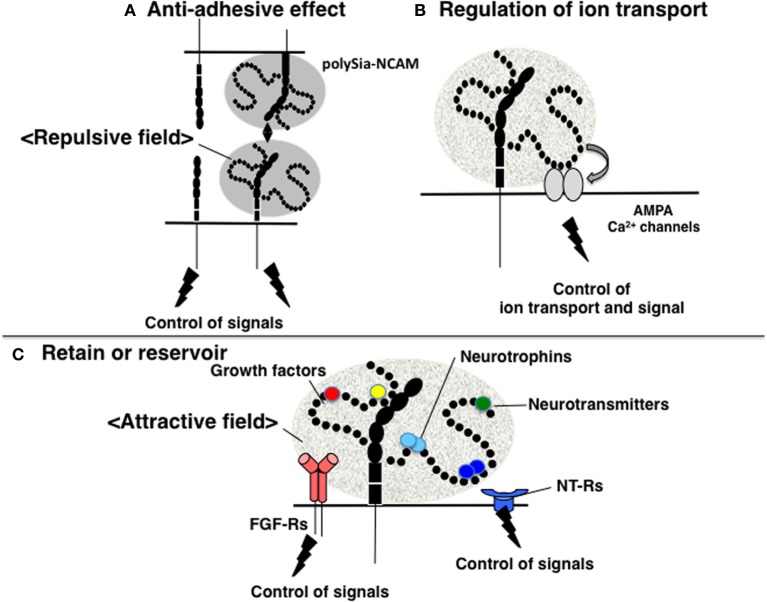
**Functions of polySia. (A)** Anti-adhesive effect. PolySia-NCAM imparts a repulsive field on the cell surface due to the large volume of polySia (shown in gray) to negatively regulate cell-cell interactions. **(B)** Regulation of ion transport. The influx and efflux of ions, such as Ca^2+^ are regulated by polySia. PolySia on NCAM interacts with Ca^2+^ channels, such as AMPA receptors, and regulates their opening and closing, by which polySia controls ion transport. **(C)** Retention or reservoir of biologically active molecules. PolySia on NCAM directly binds to various types of biologically active molecules involved in neural function, such as neurotrophins, neurotransmitters, and growth factors. PolySia-NCAM provides attractive fields that regulate their extracellular concentrations and signaling modes.

The function of polySia as a regulator of cell adhesion has been well studied in relation to NCAM. NCAM mediates not only homophilic binding, but also the heterophilic binding of other CAMs, receptors, and ECMs, including L1, Tag-1, FGF-receptor (FGFR), GFR1, collagen, HSPG, and CSPG (Gascon et al., 2007). The binding of these counterpart molecules by NCAM affects many downstream signaling pathways, including those that regulate neurite outgrowth, cell migration, fasciculation, axonal guidance and branching, and synaptogenesis. As polySia contains extremely large exclusion volumes due to its bulky polyanionic nature, polySia modification physically inhibits the homophilic binding of NCAM. Moreover, polySia-NCAM increases the intercellular space between cells, thereby inhibiting cell-cell interactions by hampering the binding between other cell adhesion molecules, as well as the docking between ligands and receptors on cell surfaces (Rutishauser, 2008). In addition to inhibiting trans-interactions, the polysialylation of NCAM may also inhibit cis-interactions with other NCAM-associated molecules on the same membrane (Gascon et al., 2007). Together, the inhibitory effects of polySia on cell adhesion are termed the “anti-adhesive effect” (Figure 3A). In addition, polySia can function as an insulating molecule because it displays the repulsive field due to the large exclusion volume.

As described above, polySia-deficient mice (ST8SIA2- and 4-DKO mice) do not survive past the early stages of development, whereas NCAM- and polySia-deficient mice (TKO mice) can typically reach adulthood. PolySia-deficiency may greatly enhance NCAM-mediated cell adhesion, which is typically suppressed by polySia during early development, leading to stronger cell adhesion and abnormal signaling. In TKO mice, the anomalous cell adhesion found in polySia-deficient DKO mice is compensated by marked reductions in cell adhesion and signal transduction due to the deficiency in NCAM. Therefore, polySia is thought to regulate cell signaling events by influencing the strength of cell adhesion (Hildebrandt et al., 2007, 2009).

To date, 27 isoforms of NCAM generated by RNA splicing have been identified, among which four major isoforms, NCAM-180, -140, -120, and sNCAM (soluble NCAM), have been characterized. NCAM-180 is the major NCAM isoform involved in the transmission of signals into cells. Among the known molecules that interact with NCAM, spectrin, Fyn, and FAK bind to the cytosolic region of NCAM-180, which contains a palmitoylation and two phosphorylation sites, while NCAM interaction with spectrin, and PKCβ2 leads to neurite outgrowth. Homophilic interactions between non-polySia-NCAMs accelerate FAK phosphorylation, recruits adaptor proteins such as Grb2, Cas, and Shc, and leads to activation of the MAP kinase pathway through Ras and Raf. In addition, non-polySia-NCAMs interact heterophilically through their extracellular domains with other CAMs (L1 and TAG1), proteoglycan (HSPG, CSPG), and FGFRs as described above. Through these heterophilic interactions, signal transduction events are not only directly regulated by NCAM, but are also indirectly regulated via other CAMs and/or growth factor receptors. All of these cell signal pathways are thus influenced by polysialylation of NCAM.

### Regulation of ion channels

Several reports have investigated the relationship between polySia and memory. For example, NCAM-KO and ST8SIA4-KO mice have impaired memory, as described above and studies on the synaptic functions through glutamate receptors have been examined. The results of *in-vitro* studies demonstrate that polySia on NCAM modulates the activity of the α-amino-3-hydroxy-5-methylisoxazole-4-propionic acid (AMPA) receptors (AMPA-Rs) in immature pyramidal neurons isolated from the CA1 region of the hippocampus (Vaithianathan et al., 2004). Specifically, polySia prolongs the open channel time of AMPA-R-mediated currents and alters the bursting pattern of the receptor channels, although polySia does not modify AMPA-R single-channel conductance (Vaithianathan et al., 2004). In this case, polySia likely directly interacts with AMPA-R (Figure 3B). In addition, there are several reports on the relationship between polySia and *N*-methyl-D-aspartate (NMDA) receptors. Impaired CA1 LTP in hippocampal slices is rescued by the addition of polySia or polySia-NCAM but not NCAM alone (Senkov et al., 2006), and polySia alone or polySia-NCAM inhibits the activation of GluN2B-containing NMDA-Rs by low micromolar concentrations of glutamate (Hammond et al., 2006). PolySia reduces the open probability, but not the conductance, of NR2B-containing NMDA-Rs in a polySia- and glutamate concentration-dependent manner by inhibiting NR2B subunit-containing NMDA-Rs through Ras-GRF1-p38 MAPK signaling cascade that is deeply involved in LTP. These findings suggest that polySia-NCAM is involved in synaptic function in the hippocampus, where it regulates different types of channels in a specific manner.

Interestingly, a unique α2,9-linked polySia structure, which was identified on the surface of sperm cells of sea urchin (Miyata et al., 2006), is involved in sperm motility through the regulation of intracellular calcium (Ca^2+^) concentrations. The regulation seems to be dependent on the binding of α2,9-linked polySia with of Ca^2+^transporters, suNCKX (K^+^-dependent Na^+^/Ca^2+^ exchanger), and suPMCA (Ca^2+^ ATPase), which are involved in regulating the influx and efflux of Ca^2+^ in sperm. Consistent with this finding, the polySia epitope has also been demonstrated to regulate sperm motility (Kambara et al., 2011). In addition to the regulation of Ca^2+^ channels, it is notable that polySia has the ability to restore Ca^2+^ ions (Shimoda et al., 1994).

### Regulator of neurologically active molecules

Recently, polySia has been shown to directly bind and regulate the function of a number of soluble bioactive factors (Sato, 2013). Thus, polySia appears to retain and regulate the function of specific bioactive factors involved in neural function in the intercellular spaces, and clearly indicates that polySia is involved in not only neurogenesis, but also in the regulation of neural function. In this case, polySia has an attractive field toward these bioactive molecules (Figure 3C). Notably, the bioactive molecules that bind to polySia have been well characterized in relation to schizophrenia and other psychiatric disorders. This novel function of polySia completely differs from its anti-adhesive effect on cell-cell and cell-extracellular matrix interactions that is mediated by its bulky nature and large exclusion volume (i.e., repulsive field), and is of particular importance to the field of the psychiatry.

#### Neurotrophins—BDNF, NT3, and NGF

Brain-derived neurotrophic factor (BDNF), which is a member of the neurotrophins, displays 50% homology with nerve growth factor (NGF) and is most abundant in brain tissue. BDNF promotes the growth and development of immature neurons, and also enhances the survival and functional maintenance of adult neurons via binding to a low-affinity receptor, p75NTR, and a high-affinity receptor, TrkB. This neurotrophic factor also has an important role in the neural plasticity that is integral to memory and learning. Notably, the analyses of NCAM-KO mice has also demonstrated that polySia-NCAM is involved in memory and learning. For example, the additions of BDNF to hippocampal slices derived from NCAM-KO mice rescued the reduction of LTP resulting from the disappearance of polySia-NCAM, indicating that polySia-NCAM is involved in signal transduction mediated by BDNF receptors (Muller et al., 2000).

**Figure 4:**
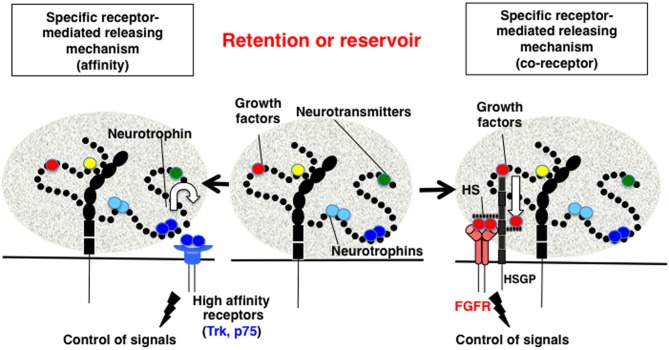
**Proposed mechanism for the retention and release of biologically active molecules by polySia. Middle panel**, PolySia captures biologically active molecules by direct binding. The retained molecules are released by one of several ways, as shown in the **left** and **right panels**. **Left panel**, Specific receptor-mediated release mechanism-1 (Affinity). In this example, brain-derived neurotrophic factor (BDNF) in a complex with polySia migrates to the BDNF receptors, TrkB or p75NTR, based on differences in the affinity between BDNF, the receptors, and polySia. **Right panel**, Specific receptor-mediated release mechanism-1 (Co-receptor). In the case of fibroblast growth factor 2 (FGF2), polySia does not release FGF2 from the FGF2-polySia complex to the FGF receptor (FGFR). Notably, FGF2 in the FGF2-polySia complex can migrate to heparan sulfate (HS) to form a FGF2-HS complex, which can then bind to FGFR as a ternary complex to enhance FGF signaling. Therefore, polySia regulates FGF2 signaling by passing FGF2 to HS and finally to FGFR.

Biochemically, the direct binding between polySia and BDNF was first demonstrated using gel filtration, horizontal native-PAGE, and surface plasmon resonance (SPR) methods (Kanato et al., 2008). These solid-based approaches were the first to demonstrate that BDNF dimers directly bind to polySia and that the minimum DP required for this interaction is 12 or greater. The resulting complex between polySia and BDNF is extremely large (approximately 2500 kDa) was shown to consist of 14 mol BDNF dimer molecules and 28 mol polySia (mean DP = 43) based on titration and gel filtration experiments. The binding of polySia is also observed with the neurotrophins NT-3 and NGF most likely through C-terminal basic regions. BDNF and polySia do not form ternary complexes with BDNF receptors and BDNF easily migrate toward receptors after forming a complex with polySia. The migration can be explained by the affinities of BDNF toward polySia and BDNF receptors (Figure 4, left panel). The *K*_D_ of BDNF toward polySia, as calculated by SPR, is approximately 10^−9^ M (Sato et al., 2010; Hane et al., 2012). In contrast, the *K*_D_ of BDNF toward TrkB and p75NTR is 10^−12^ and 10^−10^ M, respectively. Based on these results, BDNF in BDNF-polySia complexes would move toward BDNF receptors because BDNF has one to three orders of magnitude stronger affinity toward BDNF receptors than toward polySia. PolySia and polySia-BDNF complexes were also shown to increase the proliferation of neuroblastoma cells compared to untreated control cells. Recently, evidence was presented to show that ProBDNF processed outside the cell by tPA/plasmin is important for memory in the hippocampus (Pang et al., 2004). In this context, it is also important to consider the reservoir function of polySia, because proBDNF and BDNF, but not the pro-domain, are capable of binding to polySia (Sato, unpublished results). Taken together, the findings from these studies demonstrate that polySia is involved in several neurotrophin-mediated biological functions, including cell growth, neurogenesis, and memory.

#### Neurotransmitters—catecholamines

The specific binding between polySia and catecholamine neurotransmitters, particularly DA, has been demonstrated by the frontal affinity chromatography (FAC) analyses of numerous factors, including histamine, acetylcholine, serotonin, catecholamines (DA, epinephrine, and norepinephrine), and their precursors. As the binding is not observed with disialic acid (DP = 2), catecholamine binding appears specific to polySia, and it is speculated that these interactions occur between specific structures of polySia and the catechol backbone (Isomura et al., 2011). As the *K*_D_ of DA toward polySia changes depending on the pH of the solution, the specific interaction between these molecules might be fine-tuned by subtle changes of the extracellular pH (Sato et al., 2010).

PolySia is also involved in Akt signaling in human neuroblastoma cells via DRD2 (Isomura et al., 2011). It is also reported that polySia is required for DRD2-mediated plasticity involving inhibitory circuits of the rat medial prefrontal cortex (Castillo-Gómez etal., 2011). Together, these results suggest that the polySia-NCAM localized on postsynaptic membranes directly interacts with catecholamine neurotransmitters, representing a novel function of polySia.

#### Growth factors—FGF2

FGF2 is a prototypical member of the FGF family that stimulates the growth of various cell types, from fibroblasts to tumor cells, and was first identified in the bovine pituitary gland as a factor with the potential to induce fibroblast cell proliferation. FGF2 is highly expressed in the brain during earlier stages of development, and is involved in brain formation. As recent studies have demonstrated that FGF2 is a potent modulator of proliferation and differentiation of multi-potent neural progenitor cells isolated from the adult SVZ, FGF2 also plays a pivotal role in adult neurogenesis (Mudò et al., 2009). Due to its importance in both brain development and function, it is not surprising that FGF2 has been implicated in a number of psychiatric disorders (Fumagalli et al., 2005; Gaughran et al., 2006; Turner et al., 2008, 2009; Perez et al., 2009; Graham and Richardson, 2010).

FGF2-FGFR signals are enhanced following the formation of ternary complexes with heparan sulfate (HS) on HSPG, which is a component of the ECM. However, the relationship between polySia and FGF2 was not identified until several recent biochemical analyses, including gel shift assays, gel filtration, and SPR, revealed that polySia binds to FGF2 directly (Ono et al., 2012). FGF2 monomers bind to polySia and form a large complex that does not migrate toward FGFR, even if the receptors are located next to the complex. The *K*_D_ of FGF2 toward polySia (1.47 × 10^−8^ M) is smaller than that toward HS (2.81 × 10^−8^ M). Consistent with these differences in affinity, FGF2-polySia and FGF2-HS complexes display unique physical and biochemical properties. For example, FGF2-polySia binds to HS- or polySia-coated surfaces, whereas HS-polySia cannot bind to either of these surfaces, indicating that the binding regions of FGF2 to polySia and HS differ. In addition, FGF2 complexed with polySia cannot migrate toward FGFRs, but do migrate toward HS (Figure 4, right panel). It is also demonstrated that Erk and Akt signaling is regulated by polySia and HS in polySia- and HS-expressing cells, respectively (Ono et al., 2012).

## PolySia and schizophrenia

Schizophrenia is a psychiatric disorder with a complex pathophysiology that is influenced by multiple factors. It is also considered that schizophrenia is deeply related to both neurodevelopmental and neurodegenerative disorders involving disconnectivity and disorder of synapses. Indeed, there are several hypotheses such as glutamate hypothesis, DA hypothesis, and neurodevelopmental hypothesis (Coyle et al., 2012). Thus, several factors are mutually involved in pathophysiology of schizophrenia in a complicated manner, which makes it difficult to understand the underlying mechanism of schizophrenia clearly. Among the factors, polySia might be an important molecule for understanding this disorder.

Recently, indirect evidence has been reported that suggests that polySia is involved in schizophrenia. For example, it was first reported that the degree of immunostaining for polySia-NCAM derived from the hippocampus of schizophrenic brains is decreased compared with that of normal brains (Barbeau et al., 1995). In addition, chromosome 15q26, which is the genomic region containing the gene encoding ST8SIA2, is related to schizophrenia and bipolar disorders among the population of Eastern Quebec (Maziade et al., 2005). Recently, a relationship between SNPs in the promoter region of the ST8SIA2 gene and schizophrenia was identified by genome-wide association studies in the Japanese (Arai et al., 2006) and Chinese Han populations (Tao et al., 2007). The ST8SIA2 gene is also reported to be a generalized susceptibility marker for psychotic and mood disorders on chromosome 15q25-26 (McAuley et al., 2012), and is associated with an increased risk of mental illness, such as autism (Anney et al., 2010). Interestingly, the mutation of synCAM, which is another substrate for ST8SIA2, is also related with autism spectrum disorders (Zhiling et al., 2008).

As described above, to determine the relationship between genes and phenotypes, it is necessary to biochemically characterize the target gene product, particularly if it is an enzyme, because the enzymatic reaction product has a biological role. In this context, the *in-vitro* and *in-vivo* enzymatic activity of ST8SIA2 (SNP-7; Glu141Lys) that was reported from a schizophrenic patient was measured and was shown to be markedly decreased under both conditions (Isomura et al., 2011). The mutated amino acid is localized near sialyl motif L that has clearly been shown to be required for the enzymatic activity of ST8SIA2. In addition, the amount and quality (DP) of the produced polySia were also impaired (Hane et al., 2012), a result that is consistent with the histochemical data, although it only represents a change in the amount of polySia. Because the polySia structure biosynthesized by the SNP-7 of ST8SIA2 was impaired compared with that by normal ST8SIA2, the bindings toward BDNF, FGF2, and DA were also impaired (Isomura et al., 2011; Hane et al., 2012). BDNF, FGF2, and DA are known to be key molecules for the causes and biomarker of schizophrenia (Terwisscha van Scheltinga et al., 2010; Buckley et al., 2011; Balaratnasingam and Janca, 2012; Eyles et al., 2012; Tritsch and Sabatini, 2012). Impairment of the new function of polySia as a regulator of neurological active molecules will thus lead to pathophysiology of schizophrenia. Especially, behavioral deficits in psychiatric disorders have been hypothesized to arise from the elevations in the cellular balance of excitation and inhibition within neural microcircuitry (Yizhar et al., 2011). Therefore, the impact on the glycocalyx such as polySia located in the region that generates neural microcircuitry might be important. Taken together, these results suggest that changes in the quantity and quality, particularly DP, of polySia, which are closely related with the enzymatic activity of ST8SIA2, lead to an altered binding affinities toward BDNF, FGF2, and DA, may be one of the underlying causes of schizophrenia.

Anatomically, the volume of olfactory bulbs derived from schizophrenic brains is reduced (Turetsky et al., 2003), which is a similar phenotype to that of NCAM-KO mice (Cremer et al., 1994). The functional impairment and disturbed organization of the hippocampus are also involved in the etiology of schizophrenia (Harrison, 2004). In addition, a reduction of polySia-NCAM in dorsolateral prefrontal cortex of schizophrenic patients was reported (Gilabert-Juan et al., 2012). In this aspect, it is interesting that loss of ST8SIA2 or NCAM resulted in the misguidance of infrapyramidal mossy fibers and the formation of ectopic synapses in the hippocampus (Angata et al., 2004). In addition, several characteristic properties, such as brain structure, neural plasticity, and various morphological, cognitive, and emotional deficits related to schizophrenia have been observed in ST8SIA2- or ST8SIA4-SKO mice (Hildebrandt et al., 2007; Calandreau et al., 2010). Very recently, NCAM-KO mice were demonstrated that they are useful for studying specific endophenotypes with relevance to the schizophrenia although they do not display a typical schizophrenia-like phenotypes (Albrecht and Stork, 2012). As NCAM is not the only substrate for ST8SIA2 and the underlying biosynthetic mechanisms of polySia by ST8SIA2 and ST8SIA4 are not well understood, it is important to focus on the contribution of glycoepitopes, such as polySia, to schizophrenia.

## Conclusion

**Figure 5:**
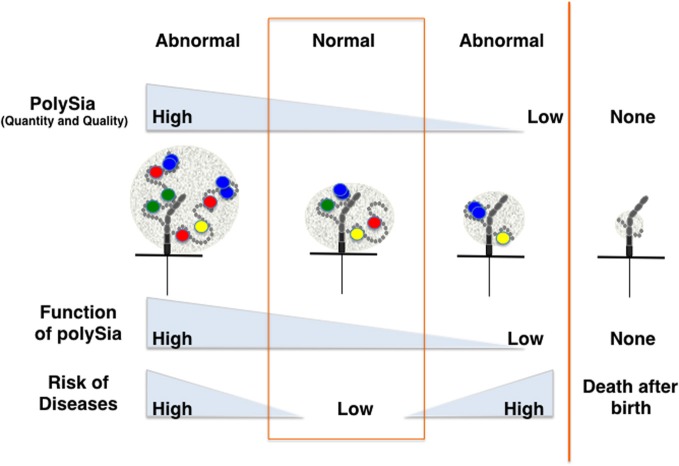
**Disease risk depends on the quantity and quality of polySia.** The quantity and quality (degree of polymerization) of polySia may differ from person to person, particularly for patients suffering from psychiatric disorders or other neurodegenerative diseases. Direct and indirect lines of evidence show that quantity and quality of polySia on NCAM are critically important for normal brain functions. Hyper- or hypo-polysialylation of NCAM might lead to the impairment of brain function through the stimulation or repression of dopamine-, BDNF- and FGF2-mediated signaling.

As psychiatric disorders such as schizophrenia are complex diseases with multiple factors contributing to pathogenesis, the mechanisms by which polySia is involved in these disorders are also likely complex. However, it is clear that the quality and quantity of polySia-NCAM are strictly regulated in normal cells, and that the impairment of polySia has profound effects on various brain functions through increasing cell adhesion, modifying ion channel activity, and reducing binding affinity toward biologically active molecules. Such impairments might lead to psychiatric disorders or affect the prognosis of these diseases (Figure 5). Interestingly, imbalances in the quantity of polySia-NCAM are also found in patients suffering from Alzheimer's disease (Mikkonen et al., 1999), Parkinson's disease (Oizumi et al., 2008), and drug abuse (Murphy et al., 2006). As demonstrated by the study of polySia, exploring the structure and function of unique glycocalyx components on the cell surface is expected to give further insight into psychiatric diseases because the glycocalyx is a major, but often ignored, player for the communication between cells and the extracellular environment.

### Conflict of interest statement

The authors declare that the research was conducted in the absence of any commercial or financial relationships that could be construed as a potential conflict of interest.
